# Cell line-specific estrogen responses uncover functional sex differences in murine macrophages

**DOI:** 10.1186/s13293-025-00760-1

**Published:** 2025-10-14

**Authors:** Alison M. Veintimilla, Zoe Turner, Nana Owusu-Boaitey, Varun Deshpande, Margaret McCarthy, Erika Moore

**Affiliations:** 1 Fischell Department of Bioengineering, University of Maryland College Park, College Park, 20742 USA; 2 School of Public Health, University of Maryland College Park, College Park, 20742 USA; 3 Department of Pharmacology and Physiology, University of Maryland School of Medicine, University of Maryland-Medicine Institute for Neuroscience Discovery, Baltimore, MD 21201 USA

**Keywords:** Macrophage function, Gonadal hormones, Estrogen

## Abstract

**Background:**

RAW 264.7 (male-derived) and J774A.1 (female-derived) cell lines are widely used in immunology research and are considered preferred models for studying signaling pathways, yet their responses to gonadal hormones remain poorly understood. Gonadal hormones, particularly estrogen, shape immune cell function and contribute to sex differences in disease outcomes, with macrophages playing a central role through their expression of intracellular estrogen receptors (ERs). Herein, we investigated ER expression and functional responses to 17β-estradiol (E2) in male-derived RAW 264.7 and female-derived J774A.1 macrophages, in 2D culture. Additionally, we looked at sex-matched and mismatched media conditions in a 3D hydrogel system. Our results reveal distinct phenotypic and functional differences between the cell lines, emphasizing the need for sex-aware approaches in immunological research and model design.

**Methods:**

RAW 264.7 and J774A.1 macrophages were cultured in basal media for 24 hours, then treated with varying concentrations of 17β-estradiol (5, 25, 100 nM), as well as hormone-free and control media. Post-treatment analyses included viability, estrogen receptor expression, phenotype skewing, matrix metalloprotease 9 (MMP9) activity, and phagocytosis. These macrophages were also used to condition sex-specific media environments and were encapsulated in a hydrogel network containing adhesive and cleavable sites. Encapsulated cells were then exposed to sex-matched or sex-mismatched conditioned media, and proliferation and MMP9 activity were assessed.

**Results:**

Our results revealed distinct differences in estrogen receptor gene and protein expression, as well as in core macrophage functions such as proliferation, inflammation, matrix remodeling, and phenotype skewing. Additionally, the sex-derivation of the surrounding molecular environment affected macrophage behavior in a 3D hydrogel system. Female-derived macrophages were more sensitive in terms of proliferation to sex-mismatched environments, while male-derived macrophages exhibited altered enzyme activity when exposed to female-conditioned media.

**Conclusions:**

These findings underscore the importance of accounting for both the origin of immune cells as well as the hormonal and environmental context in which they are studied. Without these considerations, experimental models risk missing critical biological differences that shape immune responses and disease outcomes.

**Supplementary Information:**

The online version contains supplementary material available at 10.1186/s13293-025-00760-1.

## Abstract (plain language summary)

Males and females often experience different symptoms, risks, and outcomes when it comes to certain diseases and health conditions. One reason for this may be that male and female immune cells behave differently, especially in response to hormones like estrogen. In this study, we looked at two commonly used types of mouse immune cells—one originally from a male and one from a female—to see how they respond to estrogen.

We found that male and female cells do not respond to estrogen in the same way. They showed different levels of activity, growth, and behavior depending on both the hormone exposure and the sex origin of the environment they were in. We also placed the cells in a gel that mimics tissue and exposed them to sex-specific environments and saw clear differences in how male and female cells responded. For example, female-derived cells were more sensitive in their ability to grow when placed in a “male” environment, while male-derived cells changed their behavior when exposed to signals from a “female” environment.

Our findings show that both the origin of immune cells and the environment they are placed in can strongly influence how they behave. This means that to better understand immune responses and develop more effective treatments, scientists need to consider sex as a key factor in their research models. Ignoring these differences could lead to incomplete or misleading results.

**Supplementary Information:**

The online version contains supplementary material available at 10.1186/s13293-025-00760-1.

## Highlights

Opposing regulation of *Esr1* gene and ERα protein in response to E2 across popularly used macrophage cell lines (RAW 264.7 and J774A.1).

Female- and male-derived macrophages exhibited divergent responses to increasing concentrations of E2: female-derived macrophages showed reduced phagocytic activity and inflammatory marker expression, while male-derived macrophages displayed heightened inflammatory phenotypes.

Differential regulation of MMP9, offering mechanistic insight into sex-based extracellular matrix remodeling.

Sex-mismatched media reduced proliferation in female-derived macrophages and altered MMP9 activity in male-derived macrophages within a 3D hydrogel.

**Supplementary Information:**

The online version contains supplementary material available at 10.1186/s13293-025-00760-1.

## Background

Similar to many organs in the body that exhibit sex differences in form and function, individual cell types are also influenced by sex, impacting disease progression, drug responses, and treatment outcomes [1]. Gonadal hormones and steroid hormone receptors direct sex differences associated with immune cell function. Estrogen receptors (ERs) in particular affect several key immunological regulatory pathways and processes. ER modulation impacts wound healing, autoimmunity, and viral immunity [2, 3]. ERs are intracellularly present within macrophages, a crucial innate immune cell type [4]. Macrophages are a heterogeneous immune cell population that respond strongly to environmental stimuli [5]. Estrogenic effects have been associated with macrophage self-renewal, specialization, phagocytic capabilities, immune activation, and immune tolerance [6]. The most potent estrogen-type in the human body is 17 $\beta$-estradiol (E2**)** which has been attributed to sex biases in disease outcomes through immune cell modulation [7]. While macrophages are known to express ERs, the influence of sex differences on their response to E2 and subsequent immune function has yet to be characterized. Characterization of macrophage responses to E2 is needed to create translatable experimental designs and models, especially for hormone-responsive systems.

Many in vitro studies investigating the underlying mechanisms of macrophages predominantly use the BALB/c murine cell lines, RAW 264.7 (male-XY karyotype) and J774A.1 (female-XX karyotype), to examine core functions and biophysical properties [8–10]. A PubMed database search shows that since 1980, these cell lines have been directly referenced in titles/abstracts of more than 14,000 published articles, with RAW 264.7 cells comprising nearly 80% of the total results. Additionally, RAW 264.7 cells have been referred to by the Alliance for Cellular Signaling as the preferred experimental platform for large-scale studies of signaling pathways [11]. Despite their commonplace use throughout scientific literature, these cell lines have yet to be assessed against one another in response to different hormonal challenges.

Macrophages clear pathogens and are responsible for apoptotic cell debris [12]. Subsequently, macrophages orchestrate antigen presentation and cytokine production. Macrophages drive the regulation of inflammatory responses, tissue regeneration, and downstream immune processes, including immune tolerance and chronic immune activation. Macrophages exhibit a range of phenotypic plasticity, enabling them to occupy diverse immunological roles [13]. Traditionally, their phenotypes have been broadly categorized into two functional states: pro-inflammatory and pro-regenerative [14]. Pro-inflammatory macrophages are characterized by their ability to sustain immune activation through the secretion of pro-inflammatory cytokines and chemokines, as well as the production of reactive oxygen species and nitric oxide [14]. In contrast, pro-regenerative macrophages support tissue repair and remodeling by interacting with the extracellular matrix (ECM) and facilitating the resolution of inflammation [14].

Although gonadal hormones are known to modulate macrophage behavior, many in vitro studies using macrophages fail to account for the hormonal environment and sex-related influences. How does cell karyotype (XX vs XY) influence immune behavior, and how do hormones or hormone-mimicking chemicals routinely present in cell culture conditions impact macrophage function? For example, phenol red, commonly used in media to monitor pH, is a weak estrogenic mimic whose concentration varies by vendor. These common cell culture components can have unintended variable influences on molecular outputs [15]. Additionally, sex matching experimental constituents has been demonstrated throughout the literature to influence cell–extracellular environment interactions [16–18]. Building on the importance of sex factors in experimental design, we sought to address a key gap in the literature regarding the estrogen responsiveness of commonly used macrophage cell lines.

To our knowledge, there are no prior studies that directly investigated RAW 264.7 vs J774A.1 cells’ response to E2 treatment. Here we ask how does E2 treatment and ER expression differ between these two dominantly used macrophage cell lines? We quantified ER expression at the genetic and protein level of both cell lines in response to E2 treatment. We found divergent responses between the cell lines at high concentrations of E2 at both the gene and protein level, particularly for the estrogen receptor subtype, ERα. Additionally, we assessed these cell lines’ functionalities in response to E2 treatment, characterizing proliferation, phagocytic capabilities, extracellular matrix (ECM) remodeling capabilities, inducible nitric oxide synthase (iNOS) production, and polarization.

We found that these core macrophage functions were dictated by the sex origin of the cell and E2 presence within the culture environment. We also utilized a tissue-mimetic model to assess proliferative and enzymatic activity in response to a sexed cell culture environment within a more physiologically relevant in vitro system. Specifically, we encapsulated male-derived and female-derived murine macrophage cell lines within a polyethylene glycol (PEG)-based hydrogel platform functionalized with cell-adhesive and protease-cleavable sites [19, 20]. This platform was then exposed to sex-matched and sex-mismatched culture conditions to evaluate the impact of environmental sex-secretome alignment on macrophage function. While male-derived murine macrophages were relatively unaffected by a sex-mismatched environment, female-derived macrophages exhibited reduced proliferative capacity under mismatched conditions. Additionally, male-derived macrophages cultured in a female-conditioned environment showed increased expression of the ECM enzyme MMP9, suggesting that factors within the female-derived macrophage secretome may promote an enhanced tissue remodeling environment. This study underscores key phenotypic and functional differences between macrophage immune cells of different sexes— even among routinely used immortalized cell lines.

## Materials and methods

### 2D cell culture and treating schemas

Macrophage cell lines RAWs 264.7 and J774A.1 were obtained from American Type Culture Collection (ATCC). Both lines are derived from BALB/c mice but are of different sexes with true XX and XY karyotype [9, 10]. Cells were initially cultured in Dulbecco’s Modified Eagle’s Medium (DMEM), supplemented with 10% fetal bovine serum (FBS), 100 Iµ penicillin and 100 µg/ml streptomycin (referred to in this paper as basal media; BM). Other media compositions for different treatment groups were phenol-free DMEM, with charcoal-stripped FBS, 100 µl penicillin and 100 µg/ml streptomycin (referred to as hormone free media; HF**)**. In the macrophage phenotype assessments, IFNγ (10 ng/ml) (Thermofisher) and LPS (50–100 ng/ml) (Santa Cruz Biotechnology) were added to basal media and are referred to as + IFNγ/+ LPS media. In the same aforementioned assays, IL-4 (20 µg/ml) (Prospect Bio) was added and is referred to here as + IL-4 media.

For all 2D assays, RAW 264.7 and J774A.1 cells were seeded at the same cell density ranging from ~ 5,000- 40,000 cells/cm^2 depending on the timespan, and confluency requirements for the given assay. RAW 264.7 and J774A.1 were given 24 h to adhere before being treated with different concentrations of E2 (5 nM, 25 nM, 100 nM), as well as hormone-free and basal media conditions. For phenotype assessments cells were allowed 24 h to adhere, followed by 24 h of hormonal treatment and 48 h of stimulation with + IFNγ/+ LPS or + IL-4 media.

### Synthesis of PEG-RGDS and PEG-PQ conjugates

RGDS (Arg-Gly-Asp-Ser, MW 433 g/mol; ThermoFisher Scientific) and PQ (GGGPQGIWGQGK, MW 1141 g/mol; Genscript) peptides were conjugated to acrylate-PEG-succinimidyl valerate (MW 3400 g/mol; Laysan Bio) via amine-reactive chemistry. For PEG-RGDS conjugation, RGDS and acrylate-PEG-SVA were mixed at a 1.2:1 molar ratio in 20 mM HEPBS buffer (pH 8.5) and adjusted to pH 8.0 with 0.1 M NaOH. The reaction proceeded overnight at 4 °C under gentle agitation, protected from light. The resulting acrylate-PEG-RGDS was dialyzed using a 3.5 kDa MWCO cellulose membrane (Cole-Palmer, Spectra/Por), frozen at –80 °C, lyophilized, and stored at –80 °C.For PEG-PQ-PEG conjugation, the same reaction conditions were used, with dialysis performed using a 6–8 kDa MWCO membrane. The final PEG-PQ-PEG product was similarly frozen, lyophilized, and stored at –80 °C until further use.

### 3D cell culture and treating schemas

For 3D experiments cells were cultured in T75 flasks containing basal media until reaching 40% confluency. The flask media was then replaced with hormone-free media for 24 h to allow the media to be conditioned by either RAW 264.7 or J774A.1 macrophages.

Cells were then encapsulated in a PEG-based hydrogel at a cell density of 4 × 10⁶ cells/ml. In brief, hydrogels (5 µl) were prepared using a solution of 5% w/v PEG-PQ-PEG and 3.5 mM PEG-RGDS in HEPES-buffered saline (10 mM HEPES, 100 mM NaCl, pH 7.4) containing 1.5% TEOA, 10 µM eosin Y (photoinitiator), and 0.35% N-vinyl-2-pyrrolidone (Fisher Scientific). Each hydrogel included 20,000 cells. The 5 µl hydrogel precursors were pipetted onto a polydimethylsiloxane (PDMS) slab between two ~ 380 µm PDMS spacers. A methacrylated coverslip was placed atop the setup and the mixture was polymerized under white light for 60 s. Following crosslinking, the coverslip was inverted into a 24-well plate, and 1 ml basal media was gently added.

Cells were permitted to acclimate in hydrogel conditions in basal media for 24 h. Then culture media was switched to provide sex-matched (encapsulated RAW 264.7 cells in RAW 264.7 conditioned media, encapsulated J774A.1 cells in J774A.1 conditioned media) or sex-mismatched (encapsulated RAW 264.7 cells in J774A.1 conditioned media, encapsulated J774A.1 cells in RAW 264.7 conditioned media) conditions (0.5 ml). Additional hydrogel controls continued to be cultured in basal media. Post 48 h sexed condition treatment, hydrogels were fixed and supernatant was recovered to conduct immunocytochemistry (ICC) and soluble factors analysis.

### Metabolic assay in response to E2 treatment

A resazurin-based metabolic assay was performed per manufacturer instructions (ThermoFisher, cat. no. A50101) post E2, basal media and hormone free treatment. Additional cytotoxic controls were plated with media that contained 10% dimethyl sulfoxide (DMSO). Blank wells including either basal media or hormone-free media were included to normalize fluorescence readings. In brief, 10 µl of resazurin reagent was added per well and allowed to incubate for 2.5 h. Fluorescence of the plate was then read on a microplate reader (Spark, Tecan) at 560 nm excitation and 590 nm emission.

### Immunocytochemistry of proliferation marker Ki67 and ERα

The time and antibody dilution ranges are referencing 2D vs 3D staining. Following 24 h of hormone treatment or phenotypic skewing, cells and hydrogels were fixed in 4% paraformaldehyde for 20 or40 minutes at room temperature, then washed 3 × with Tris-buffered saline (TBS). Cells were permeabilized with 0.125 −0.25% TritonX for 10–45 min at room temperature and then washed 4 × with TBS (5 min per rinse). Cells were subsequently blocked with 5% donkey serum for 2 h-overnight at 4 °C. After blocking, cells were incubated at 4 °C with primary antibodies for Ki67 (rabbit-anti-mouse polyclonal antibody, Abcam, ab15580) at a 1:200 or 1:400 dilution in 0.5% donkey serum overnight-1 day. For ICC of ERα primary rabbit-anti-mouse polyclonal antibodies (Invitrogen, cat. no. PA1-309) were utilized at a 1:400 dilution in 0.5%. After primary incubation, cells were rinsed 4 × with TBS + 0.01% Tween for 2 or 6 h, with the final rinse being just TBS. Cells were then incubated at 4 °C with secondary antibody Alexa Fluor 555 (Invitrogen, A31570) or Alexa Fluor 488 (Thermofisher, A-21206) at a 1:400 dilution overnight-1 day. Cells were then given a TBS rinse for 1–3 h. DAPI nuclear stain was added to cells for an hour-overnight following 3 × TBS washes (5 min each).

Imaging was then done using Andor BC43 benchtop confocal for DAPI (blue channel), Ki67 (red and green channel), and ERα (green channel). One image per well was chosen randomly for four wells per condition for 2D studies. Image analysis was conducted through automatic thresholding (Ki67) or Cellpose ROI quantification (ERα) for DAPI/cell count. Then a positive signal was counted via manual marking (ERα)/automatic thresholding (Ki67) and subsequent particle analysis function on Fiji ImageJ. Imaging within the hydrogel was taken as Z-stacks with 1.5 µm and a range of 40 µm. Hydrogel Z-stacks were imported into IMARIS, and cells were identified using spot analysis of the DAPI channel. The Imaris machine learning algorithm was then trained to identify DAPI (blue) positive cells that also expressed Ki67 (green). The output of the algorithm was checked for accuracy, and the number of Ki67 positive and negative cells was then quantified.

### Phenotypic morphology assessment

To conduct these assessments cells were fixed and stained for DAPI post hormonal pre-treatment and phenotypic skewing. Differential phase contrast imaging was then performed for all treatment wells (4 wells per group condition) using Andor BC43 benchtop confocal microscope. Cellpose algorithm for cellular segmentation was then trained to generate ROIs and consequent aspect ratio. ROIs were imported to Fiji ImageJ and manually checked for accuracy. The first 50 accurately generated/manually redrawn aspect ratio measurements were taken per well. Aspect ratio is defined as the ratio of the cell’s major axis to its minor axis, as described in previous studies [21, 22]. Statistical analysis was run on well averages as technical replicates, so as to not falsely power analysis. Singular measurements to demonstrate distribution of aspect ratio measurements are offered in supplementary documentation.

### Reverse transcriptase quantitative polymerase chain reaction (RT-qPCR) for Esr1, Esr2, arg-1, and iNOS

Following E2, basal media, hormone-free treatments, or phenotype skewing (treatment with + IL-4 or + LPS/IFNγ media) cells were lysed and total RNA was extracted using the RNeasy Mini Kit (Qiagen, 74,104), then quantified with a NanoDrop spectrophotometer. Reverse transcription was performed with 50–100 ng of total RNA using the iScript cDNA Synthesis Kit (Bio-Rad). Complementary DNA was amplified using SYBR Green PCR Master Mix (Bio-Rad). A pre-validated SYBR Green primer pair for mouse *Esr1*, *Esr2*, *Arg-1*, and *iNOS* were used according to the manufacturer instructions (refer to table below). The cycling parameters were as follows: 95 °C for 25 s (polymerase activation and DNA denaturation), 95 °C for 2 s (denaturation), and then 60 °C for 25 s (annealing/extension) for a total of 40 cycles. Relative gene expression was calculated using the 2^(-ΔΔCT) method, with β-actin as the internal control and the basal media treatment group as the calibrator.

**Table:** 

Gene symbol	Assay description	Vendor	Catalog number	Notes
*Esr1*	PrimePCR SYBR Green Assay (mouse)	Bio-Rad	qMmuCED0044294	Pre-validated assay
*Esr2*	PrimePCR SYBR Green Assay (mouse)	Bio-Rad	qMmuCID0005566	Pre-validated assay
*Arg1*	PrimePCR SYBR Green Assay (mouse)	Bio-Rad	qMmuCID0022400	Pre-validated assay
*iNOS*	Custom Primers	IDT	fwd: 485010148 rev: 485010147	Custom order 5’−3’ fwd: TTT GCT TCC ATG CTA ATG CGA AAG rev: GCT CTG TTG AGG TCT AAA GGC TCG
*Actb*	PrimerPCR SYBR Green Assay (mouse)	Bio-Rad	qMmuCEP0039589	Pre-validated assay

### Phagocytosis assay in response to E2 treatment

RAWs 264.7 and J774A.1 were given 24 h to adhere before being treated with different concentrations of E2 (5 nM, 25 nM, 100 nM), as well as hormone-free and basal media. Blank wells with either basal or hormone-free media. Phagocytosis was then assessed through Invitrogen pHrodro Red E. coli BioParticle™ conjugates (P35361) per manufacturer instructions. Cells were incubated with bioparticles for 2 h post-treatment period and subsequent fluorescence intensity was measured using an excitation and emission maxima of 560 nm and 585 nm respectively on a microplate reader (Spark, Tecan). Phagocytic capacity was then calculated through the subtraction of background fluorescence reads from experimental wells.

### Zymography for MMP9 in response to E2 treatment and sex-matched/mis-matched environments

Conditioned media from RAW 264.7 and J774A.1 cells following treatment with E2 (5 nM, 25 nM, 100 nM), hormone-free, basal, or phenotype-specific media were collected and stored at –80 °C until analysis. Supernatants from hydrogel encapsulation experiments were similarly collected and frozen at –80 °C. Protein concentration was determined using a bicinchoninic acid (BCA) protein assay following the manufacturer’s instructions (Thermo Scientific, 23,225). Absorbance was measured at 562 nm using a microplate reader (Spark, Tecan). The average absorbance of each sample and standard was calculated, and a linear regression was applied to the BSA standard curve. The resulting equation was used to interpolate protein concentrations of experimental samples.

Gelatin zymography was used to assess MMP9 activity in the conditioned media. A total of 20 μg of protein per sample, as determined by BCA assay, was mixed with non-reducing sample buffer and loaded onto 10% zymogram gelatin gels (Novex, ZY00105BOX). A molecular weight protein ladder (Thermo Fisher Scientific, 26,623) and 125 ng of recombinant mouse MMP9 protein (Abcam, ab39309) were included on each gel as size and activity controls, respectively. Gels were run at 125 V and 0.03 mA for approximately 120 min at 4 °C in 1X Tris–Glycine SDS running buffer (Novex, LC2675). Following electrophoresis, gels were incubated in 1X renaturation buffer (Novex, LC2670), followed by 1X development buffer at room temperature for 30 min each with gentle agitation to remove SDS and restore enzyme activity. Gels were then transferred to 1X developing buffer (Novex, LC2671) and incubated at 37 °C overnight to allow substrate digestion.

After incubation, gels were washed twice in deionized water for 15 min each, stained for 1 h using Imperial™ Protein Stain (Thermo Fisher Scientific, 24,615), and subsequently washed twice in deionized water for 1 h each, until clear bands representing proteolytic activity were visible. Gels were imaged using LI-COR Odyssey CLx at 169 µm resolution, medium scan quality, 0.5 intensity, and 0.5 mm focus offset range. Image analysis was performed in ImageJ. Lanes were defined and plotted using the gel analyzer tool, and the area under the curve (AUC) was measured for each unstained band to quantify relative MMP9 activity. AUC values were normalized to the recombinant mouse MMP9 control band intensity.

### Enzyme-linked immunosorbent assay (ELISA) for inducible nitric oxide synthase (iNOS) production

A colorimetric sandwich ELISA for mouse iNOS (Abcam, ab253219) was performed per manufacturer instructions. Supernatants from phenotype studies were diluted in 1X cell extract buffer to 300 µg/ml, respectively, with protein concentrations determined by BCA (see Zymography section). Standard mouse iNOS was serially diluted from 3,000 to 46.88 pg/ml, including a blank (0 pg/ml), and plated in duplicate; samples were plated in triplicate. A capture/detection antibody cocktail was added to all wells, followed by a 1 h incubation at room temperature on a plate shaker (400 rpm). After three washes with Wash Buffer PT, tetramethylbenzidine substrate was added and incubated for 5–10 min at room temperature on a plate shaker (400 rpm). Stop solution was then applied for 1 min, and absorbance was measured at 450 nm using a microplate reader (Spark, Tecan).

### Statistical analysis

Throughout ICC experiments, DAPI + cell counts were exported into GraphPad Prism 10.1.1 (La Jolla, CA). All cell counts were ensured to be non-significant between treatment groups within a given cell line. In 2D experiments, sampling from wells were considered as technical replicates. There were always 3–4 technical replicates per condition for all assays. In 3D experiments, 3 hydrogels were fabricated per condition and considered technical replicates. A one-way analysis of variance (ANOVA) with a post-hoc Tukey test was conducted between hormone-treatment and sex-matching treatment groups. Statistical significance is reported as *p < 0.05, **p < 0.01, and ***p < 0.001. For all plate reader assays coefficient of variation was calculated and ensured to be no greater than 15%. Results are presented as the mean ± standard deviation.

## Results

### E2 treatment had no effect on viability but differentially regulated estrogen receptor expression in RAW 264.7 and J774A.1 cells

**Fig. 1:**
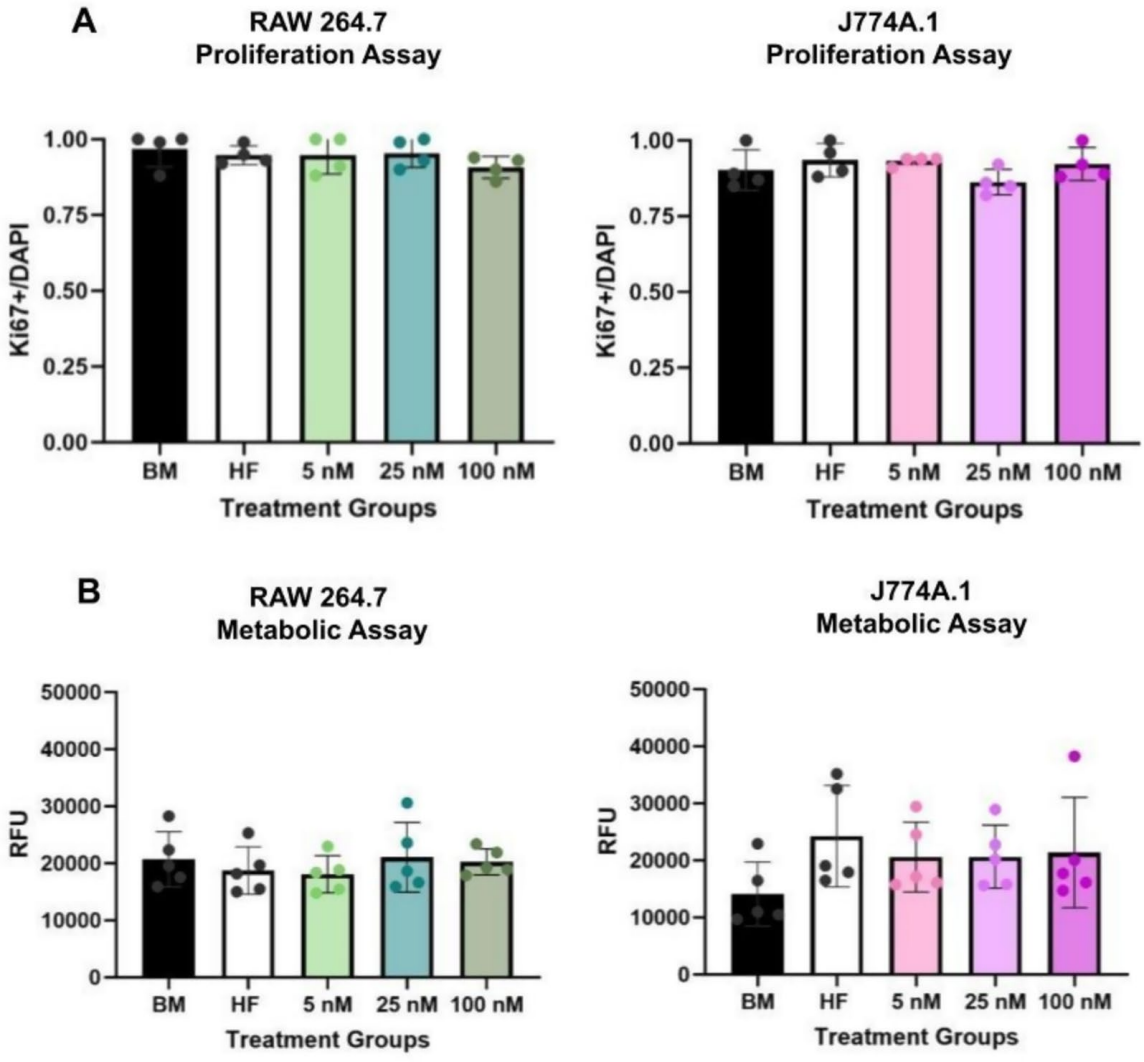
There are no major differences in viability after E2 dosing for RAW 264.7 (male-derived) and J774A.1 (female-derived) macrophage cell lines. **A** Ki67 to DAPI ratios for RAW 264.7 (left) and J774A.1 (right) based on immunocytochemistry. There were 4 technical replicates. **B** Resazurin metabolic assay relative fluorescent units (RFU) results for RAW 264.7 (left) and J774A.1 (right) cells with 5 technical replicates per groups. For figure, BM refers to basal media conditions, HF refers to hormone-free conditions and 5, 25,100, refers to E2 treatment in nM. One-way ANOVA and post hoc Tukey statistical analysis conducted. Bars within the graph are demonstrating ± SD

To assess cell viability in response to E2 treatment, we measured metabolic activity using resazurin reduction and proliferation via Ki67 expression. There were no statistically significant differences in either metabolic activity (Fig. 1A) or proliferative capacity (Fig. 1B, Supplement 1 A) between RAW 264.7 and J774A.1 under varying hormonal conditions.

**Fig. 2:**
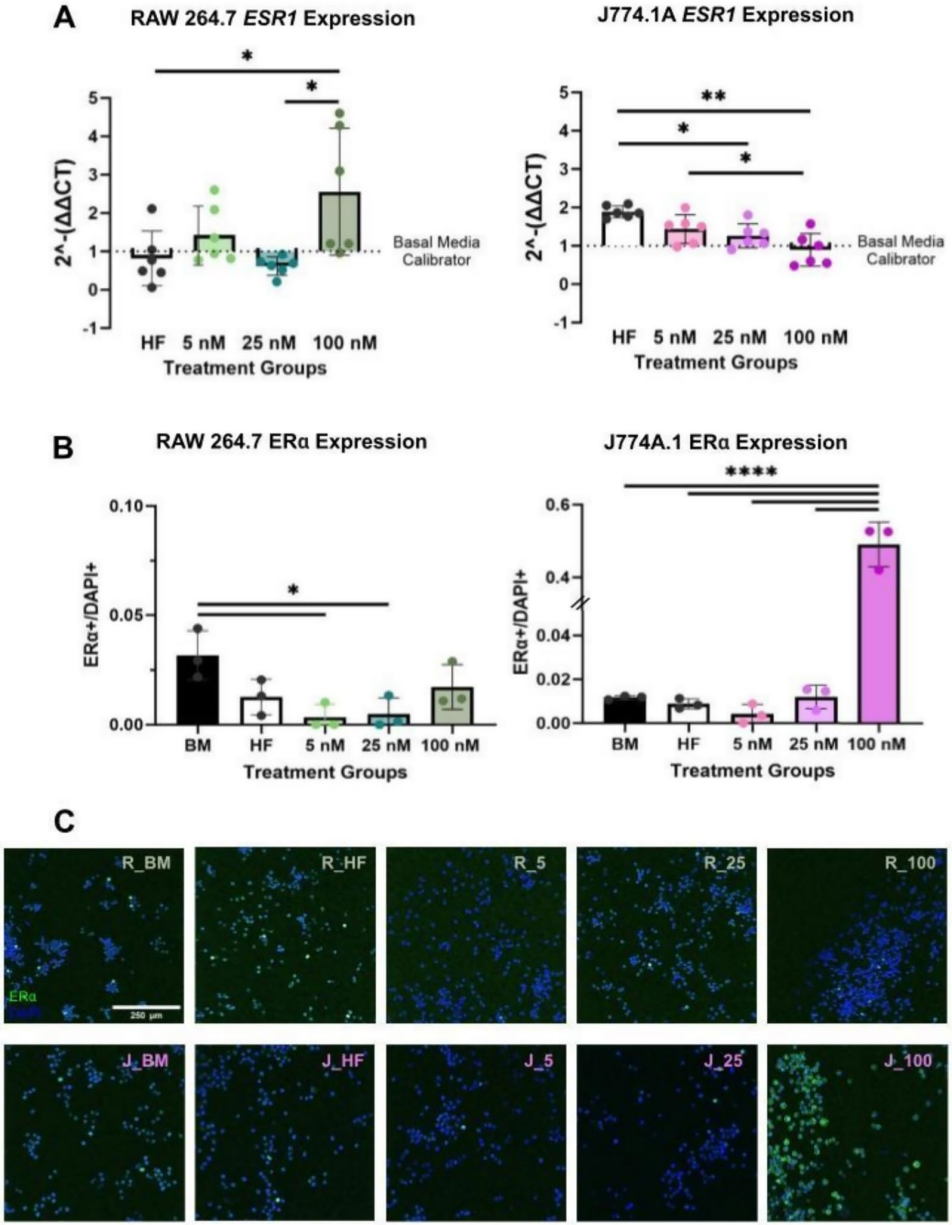
Differential ER expression at a high E2 concentration for RAW 264.7 vs J774A.1. Gene expression of *Esr1* at high E2 (100 nM) dosage for RAW 264.7 vs J774A.1. J774A.1 cells exhibit significantly higher expression of ERα compared to RAW 264.7 cells under a high concentration E2 treatment, as well as relative to other hormonal treatment conditions. **A** *Esr1* RT-qPCR quantification for RAW 264.7 (left) and J774A.1 (right) in response to varying E2 dosages with basal media group as calibrator (6 technical replicates). **B** ERα to DAPI ratios based on immunocytochemistry with 3 technical replicates. Double slashes (//) in the figure refers to a value jump in the y-axis. **C** Representative images of ICC staining for ERα (green), and cell-nuclear marker, DAPI (blue) on RAWs 264.7 (top row) and J774A.1 s (bottom row). Scale bar at 250 μm. For all – BM is referring to basal media conditions, HF is referring to hormone-free conditions and 5, 25, 100, refers to E2 treatment in nM. One-way ANOVA and post hoc Tukey statistical analysis conducted (*p < 0.05,**p < 0.01,***p < 0.001, ****p < 0.0001). F-values for **A** (4.72, 9.103), and **B** (5.055, 182.4) were > 1. Bars within the graph are demonstrating ± SD

Expression of *Esr1* and *Esr2*—encoding estrogen receptor subtypes α and β, respectively—was quantified via RT-qPCR following E2 treatment. Interestingly, RAW 264.7 (male-derived) and J774A.1 (female-derived) cells displayed divergent *Esr1* gene expression responses to a high concentration (100 nM) of E2. At a high E2 concentration (100 nM), RAW 264.7 cells showed a significant ~ 3 × increase in *Esr1* expression (p = 0.0291), whereas J774A.1 cells exhibited a significant ~ 1 × decrease (p = 0.0003) compared to hormone-free conditions (Fig. 2A). *Esr2* expression was also measured in response to E2 but the cycle threshold (CT) values were > 35 indicating low to undetectable expression (Supplement 1B). This is in line with other studies reporting these cell lines do not express ERβ when exposed to similar E2 concentrations such as the ones used in this study [23].

Given the results of gene expression analysis, immunocytochemistry (ICC) was conducted for ERα. Notably, while mRNA for *Esr1* was high for RAW 264.7, protein expression of ERα was relatively low across E2 treatment groups (Fig. 2B**, **Fig. 2C). Similarly, J774A.1 macrophages demonstrated a significantly high (p < 0.0001) expression of ERα at a high E2 concentration compared to all other treatment groups, opposing aforementioned gene (mRNA) expression results (Fig. 2B**, **Fig. 2C). These observations are likely due to the tightly regulated nature of ERs.

### E2 treatment has divergent effects on macrophage functional capabilities in a sex-dependent manner

**Fig. 3:**
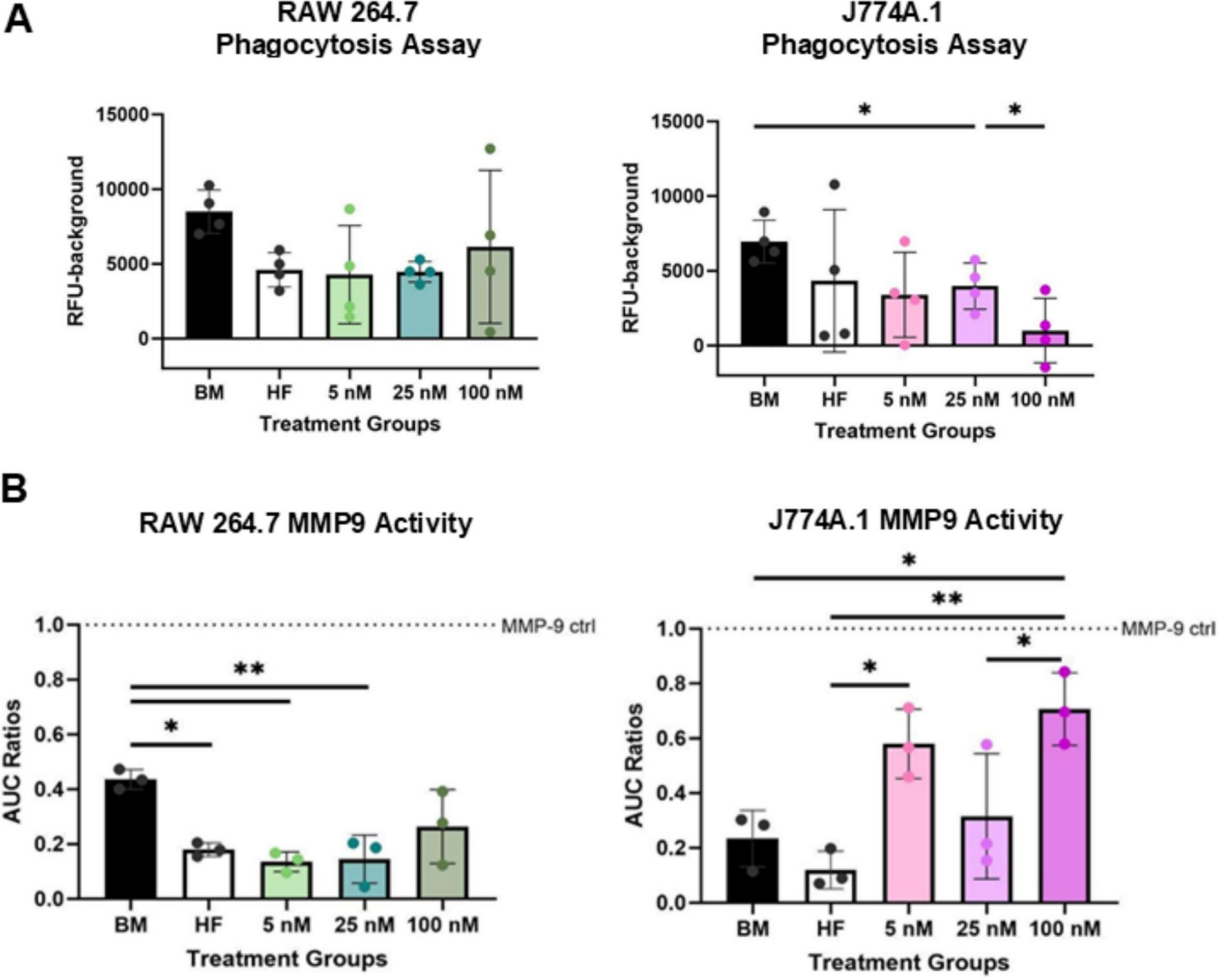
E2 has divergent effects on RAW 264.7 vs J774A.1 s’ functions. **A** RFU values of pHrodro bioparticle phagocytosis assay for RAW 264.7 (left) and J774A.1 (right) with 4 technical replicates per vendor instruction. **B** Zymmography analysis for ECM remodeling enzyme MMP9 for RAW 264.7 (left) and J774A.1 (right) (3 technical replicates). The area under the curve (AUC) ratios were done based on an isolated MMP9 control. For figure, BM refers to basal media conditions, HF refers to hormone-free conditions and 5, 25, 100, refers to E2 treatment in nM. One-way ANOVA and post hoc Tukey statistical analysis conducted (*p < 0.05,**p < 0.01). F-values for **A** (1.553, 2.285) and **B** (8.041, 8.867) were > 1. Bars within the graph are demonstrating ± SD

Phagocytic activity in both cell lines was assessed using pH-responsive, fluorescently conjugated E.coli beads that emit fluorescence upon acidification within macrophage endosomes. RAW264.7 and J774A.1 cells experienced similar reductions to 40–50% effect in their ability to phagocytize within hormone-free, 5 nM, and 25 nM E2 treatment groups (Fig. 3A). Most notable were the responses to the 25 nM E2 treatment, which significantly (p < 0.05) depleted phagocytic capabilities in J774A.1 cells (Fig. 3A), relative to basal media conditions. There were non-significant effects on phagocytosis noted with RAW 264.7 when the surrounding microenvironment was supplemented with E2. These results suggest that at an intermediate concentration of E2 demonstrates inhibition of J774A.1 cells’ phagocytic abilities.

In our other functional assessment, we demonstrated that MMP9 production in response to E2 dosing had a greater effect on J774A.1 cells compared to RAW 264.7 cells. In RAW 264.7 cells, MMP9 levels were reduced in hormone-free media compared to basal media conditions. E2 supplementation did not restore MMP9 production, regardless of concentration (Fig. 3B). In contrast, J774A.1 cells exhibited a concentration-dependent response to E2, with significant increases in MMP9 production at both low (5 nM, p = 0.0177) and high (100 nM, p = 0.0035) E2 concentrations relative to hormone free conditions, but a notable decrease at an intermediate concentration (25 nM) (Fig. 3B). However, the intermediate concentration remained elevated relative to hormone-free and basal media conditions, though the difference was not statistically significant. These divergent responses suggest potential estradiol and sex-based differences in ECM remodeling capacity.

### E2 pre-treatment of RAW264.7 and J774A.1 macrophages impacts inflammatory phenotype skewing

**Fig. 4:**
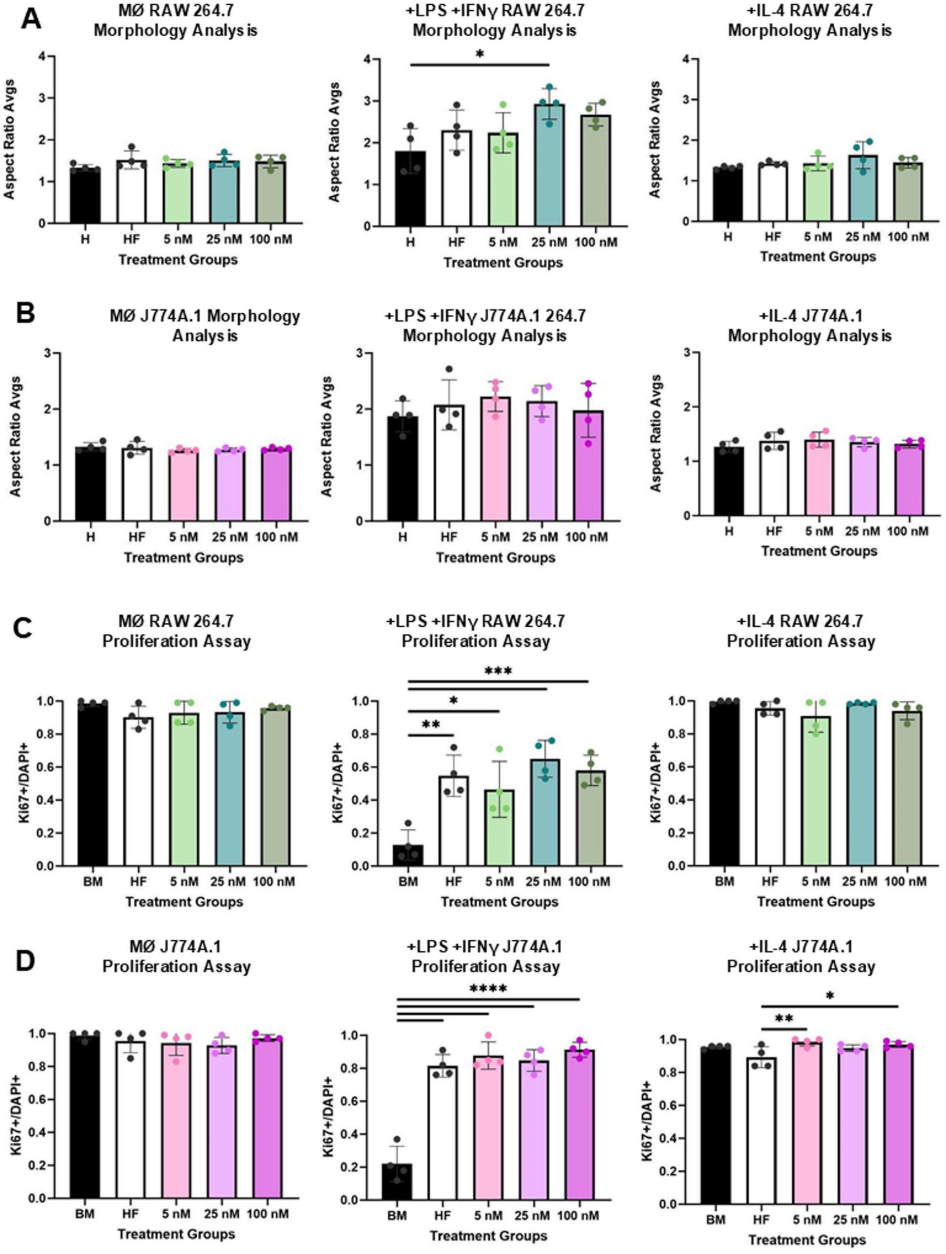
E2 enhances elongation and proliferation in + LPS + IFNγ activated macrophages. RAW 264.7 macrophages pre-treated with increasing concentrations of E2 exhibit a more elongated morphology following + LPS + IFNγ activation. Both RAW 264.7 and J774A.1 macrophages show increased proliferation in response to E2 pre-treatment under inflammatory conditions. Aspect ratio morphology analysis for RAW 264.7 (**A**) and J774A.1 (**B**). Ki67 and DAPI ratios based on immunocytochemistry for RAW 264.7 (**C**) and J774A.1 (**D**) cells. For figure, + LPS + IFNγ refers to an inflammatorily skewed macrophage post E2 treatment. + IL-4 refers to a pro-regenerative skewed macrophage post E2 treatment. There were 4 technical replicates per group for **A-D**. BM refers to basal media conditions, HF refers to hormone-free conditions and 5, 25,100, refers to E2 treatment in nM. One-way ANOVA and post hoc Tukey statistical analysis conducted (*p < 0.05,**p < 0.01,***p < 0.001). For RAW 264.7 cells in **A**, the F-value was above 1 (3.906), whereas for J774A.1(**B**) it was below 1 (0.589), with no significant effects being drawn for J774A.1. F-values for **C** (11.31) and **D** (57.47, 4.498) phenotypes that demonstrated significant comparisons were > 1. Bars within the graph are demonstrating ± SD

Following E2 pre-treatment both cell lines were polarized toward pro-inflammatory (+ LPS + IFNγ) or pro-regenerative (+ IL-4) phenotypes and evaluated for proliferation, morphology, gene expression, iNOS, and MMP9 production. Morphological data suggests a trending increase in cellular aspect ratio in response to E2 pre-treatment for + LPS + IFNγ skewed RAW 264.7 macrophages (Fig. 4A-B, Supplement 2). Inflammatory RAW 264.7 macrophages demonstrated a statistically significant increase in aspect ratio at an intermediate E2 dosage (25 nM, p = 0.014) when compared to basal media condition (Fig. 4A-B). Both cell lines showed similar increases in Ki67 expression with rising E2 pre-treatment when differentiated into the + LPS + IFNγ–induced inflammatory macrophage phenotype. (Fig. 4C-D).

**Fig. 5:**
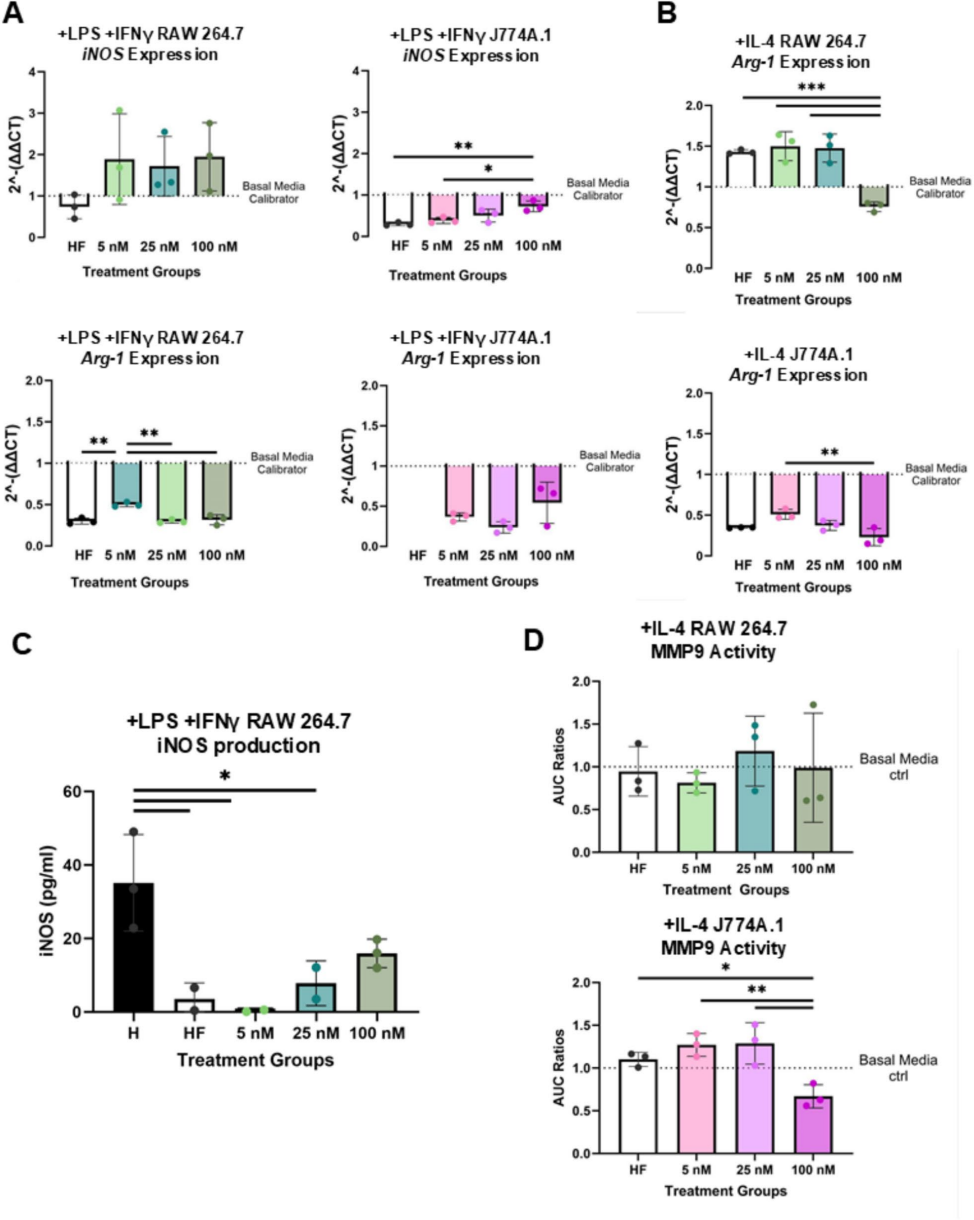
RAW 264.7 and J774A.1 macrophages differ in gene expression of canonical phenotypic markers and production of phenotypic enzymatic proteins. **A-B** RT-qPCR quantification of phenotypic markers (*iNOS, Arg-1*) for RAW 264.7 (left/top) and J774A.1 (right/bottom) post phenotypic stimulation (+ LPS + IFNγ or + IL-4 media treatment) in response to varying E2 pre-treatment. **C** Detectable quantification of iNOS production in + LPS + IFNγ RAW 264.7 macrophages. **D** MMP9 Expression via zymography for + IL-4 RAW 264.7 (top) and + IL-4 J774A.1 (bottom) macrophages in response to E2 pre-treatment. For figure, **A-D** 3 technical replicates were used. Additionally, BM refers to basal media conditions, HF refers to hormone-free conditions and 5, 25,100, refers to E2 treatment in nM. One-way ANOVA and post hoc Tukey statistical analysis conducted (*p < 0.05,**p < 0.01,***p < 0.001). F-values for **A**-**D** phenotypes that demonstrated significant comparisons were > 1, ranging from 1.5–25. Bars within the graph are demonstrating ± SD

Gene expression of the canonical inflammatory marker *iNOS* was non-significantly (p > 0.05) upregulated in + LPS + IFNγ RAW 264.7 cells, and significantly (p = 0.0057) upregulated in + LPS + IFNγ J774A.1 cells at 100 nM E2 compared to the hormone-free condition (Fig. 5A). On the other hand, *Arg-1* expression (canonical pro-regenerative marker) was significantly (p = 0.0011) increased for + LPS + IFNγ RAW 264.7 in the presence of 5 nM E2 when compared to hormone free conditions (Fig. 5A). No *Arg-1* expression was detected in + LPS + IFNγ J774A.1 cells for the hormone free condition. Both + IL-4 RAW 264.7 and + IL-4 J774A.1 macrophages exhibited decreasing expressions of *Arg-1* with increasing E2 pre-treatment (Fig. 5B). Cycle threshold (CT) values of *iNOS* for + IL-4 and MØ macrophages were >/= 35 or undetermined (Table Supplement 1). CT values for *Arg-1* in MØ macrophages were also >/= 35 or undetermined (Table Supplement 2).

We quantified iNOS production and MMP9 activity using ELISA and zymography, respectively. iNOS protein production in + LPS + IFNγ RAW 264.7 s demonstrated a significant decrease (p < 0.05) in hormone free (p = 0.0013), 5 nM E2 (p = 0.0008), 25 nM E2 (p = 0.0025) conditions compared to basal media conditions (Fig. 5C). At 100 nM E2, iNOS was restored to levels that were not significantly (p = 0.1040) different from the hormone-free condition (Fig. 5C). Production of iNOS by + LPS + IFNγ J774A.1 was below the assay’s detection limit for all conditions. + IL-4 J774A.1 demonstrated a significant (p < 0.05,0.01) decrease in MMP9 production at 100 nM E2 relative to all other condition groups (Fig. 5D). + IL-4 RAW 264.7 macrophages had no significant differences in MMP9 production across different treatment groups (Fig. 5D).

These data suggest that E2 treatment impacts macrophage phenotypic function in a sex-based manner.

### Sex-matched media environments critically influence macrophage proliferation, inducible nitric oxide synthase, and MMP9 production in a 3D tissue mimetic hydrogel

**Fig. 6:**
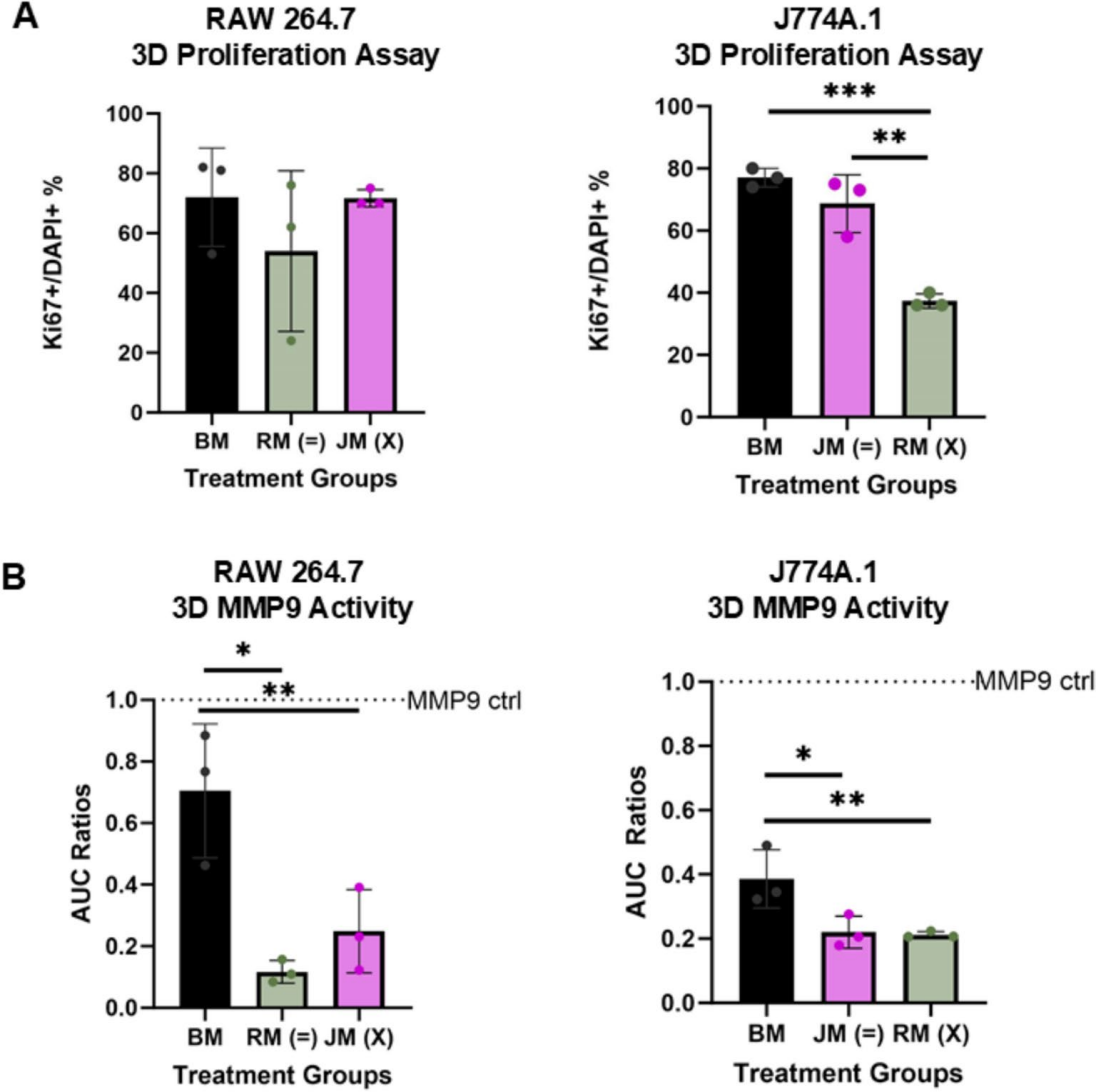
Sex-mismatched secretomic environments differentially influence macrophage behavior in 3D culture. **A** Ki67 + over DAPI + ratios based on immunocytochemistry imaging for RAW 264.7 (striped) and J774A.1 (dotted) in sex-matched and sex mis-matched environments. **B** MMP9 expression via zymography for RAW 264.7 (striped) and J774A.1 (dotted) in sex-matched and sex mis-matched environments. Three hydrogels were used per group throughout the figure. For figure – BM refers to basal media, RM (green) refers to RAW 264.7 conditioned media, JM (pink) refers to J774A.1 conditioned media. One-way ANOVA and post hoc Tukey statistical analysis conducted (*p < 0.05,**p < 0.01,***p < 0.001). F-values for **A-B** were for relevant comparisons > 1 (2.5–39). Bars within the graph are demonstrating ± SD

RAW 264.7 and J774A.1 macrophages were encapsulated in a tissue-mimetic hydrogel platform and exposed to sex-matched/sex-mismatched environments. Encapsulated cells were evaluated in terms of proliferative capacity, MMP9, and iNOS production. RAW 264.7 macrophages exhibited no significant differences in Ki67 expression across groups (Fig. 6A). J774A.1 cells displayed a 30–40% decrease in Ki67 expression in a sex mis-matched environment compared to sex-matched (p = 0.0014) and basal media conditions (p = 0.0004) (Fig. 6B)**.** Additionally, MMP9 production was non-significantly elevated in male-derived macrophages exposed to sex-mismatched female-derived-conditioned media, despite baseline MMP9 levels in the conditioned media itself showing no significant difference between sexes (Supplement 3). These findings suggest that sex-specific cell-secretome interactions may play a critical role in regulating macrophage proliferation and ECM remodeling capacity.

## Discussion

Incorporating sex-accurate environments and sex-relevant cells in disease modeling, therapeutic assessments, and diagnostic development is essential for achieving clinically translatable technologies. However, sex is often overlooked as a variable in preclinical experimental design, significantly affecting downstream workflows. This oversight can lead to costly delays and inefficiencies, in terms of safety, time, and resources. With this in mind, we investigated the influence of sex on immune cell function and found that it significantly affects macrophage responsiveness to E2 and to sex-specific secretome environments. As key regulators of inflammation and tissue repair throughout the body, macrophages are a crucial cell type in immunological studies. Herein, we sought to thoroughly characterize the different sex-based effects on RAW 264.7 (male-derived) and J774A.1 (female-derived) cell lines in estrogen receptor (ER) expression, proliferative, functional, and phenotypic capabilities.

In this study, we observed differential expression between mRNA and protein levels for ERs. Specifically, upregulation of *Esr1* mRNA expression coincided with reduced ERα protein levels, while downregulation of *Esr1* was associated with increased ERα protein expression. Notably, J774A.1 (female) macrophages exhibited substantially higher ERα protein expression but had significantly downregulated *Esr1* at high concentrations of 17β-estradiol (E2). This suggests that estrogen-mediated pathways are tightly regulated, possibly to mitigate the carcinogenic effects of estrogen [24, 25]. This regulation may reflect the macrophage cell’s attempt to maintain homeostasis, with higher protein expression occurring alongside mRNA downregulation within a specific timeframe. This pattern aligns with literature indicating that increased ERα binding activity coincides with *Esr1* downregulation, akin to the action of tamoxifen, a well-known ER antagonist [26]. Interestingly, RAW 264.7 ERα protein expression in general maintains relatively low signal across all treatment groups. In macrophages specifically, heightened ERα expression has been linked to intensified inflammatory responses [27]. Consequent divergent behaviors (especially regarding inflammatory pathways) between RAW 264.7 and J774A.1 macrophages in response to ERα ligand, E2, can therefore, in part, be attributed to differences in mRNA and protein ER quantities.

In this work, phagocytic capabilities appeared to be both hormone- and sex-dependent. Significant effects and mediation were only observed to influence J774A.1 macrophages. These data suggest that J774A.1 phagocytic capacity is more greatly impacted by ER modulation relative to RAW 264.7. The effects on J774A.1 cells also align with previous studies displaying decreased tumoricidal phagocytosis in the presence of E2 [13]. Previous studies have reported an inhibition of J774A.1 cell phagocytosis in an E2 concentration-dependent manner [28].On a broader perspective, E2 sensitivities on phagocytic capacity in female-derived macrophages (J774A.1) could lead to more pathogens and debris in the body, in turn causing persistent bodily inflammation in response to these materials [13, 28]. This may play a role in a higher prevalence of certain chronic diseases in women, a result of naturally higher circulating and fluctuating levels of E2 in women compared to men [29].

E2 and sex-based factors may also influence macrophage-mediated remodeling of the ECM. In this study, high (100 nM) and low (5 nM) E2 supplementation both elevated MMP9 activity in J774A.1 cells compared to basal and hormone-free conditions. Interestingly, at an intermediate E2 concentration (25 nM), this effect was less pronounced and significantly lower than at the high E2 concentration. These findings suggest that E2 exerts a concentration-dependent, bimodal effect on female-derived macrophages. MMPs are key biomarkers of macrophage-mediated remodeling of the extracellular environment. Among them, MMP9 is particularly important, as its dysregulated activity is implicated in both physiological and pathological remodeling processes [30]. Increased MMP9 activity has been linked to increased inflammation, fibrosis, and cardiovascular complications [30–32]. These findings, therefore, may contribute to a broader understanding of the mechanistic basis for sex-based differences observed in cardiovascular disease.

Macrophage phenotype skewing is critical for macrophage niche fulfillments at given tissue sites. Baseline characterization of macrophage morphology, proliferation, and mRNA expression, along with subsequent assessments of enzyme activity and nitric oxide production, enables the identification of macrophage phenotypic skewing as both sex-dependent and influenced by the presence of E2. By analyzing proliferative and morphological outcomes, we established baseline characterizations of sex-specific, phenotypically skewed macrophage cell lines following E2 pre-treatment. Geometric descriptors of cell shape, such as aspect ratio, provide insight into cell elongation, division mechanics and interdispersion, gene expression, signaling pathways, and adhesion properties [33–35]. We utilized aspect ratio as a quantitative proxy to cell elongation, proliferation and dispersion dynamics. Macrophage elongation, regulated by cytoskeletal dynamics and microenvironmental cues, underlies polarization and phenotype switching between pro-regenerative and proinflammatory states. Our aspect ratio analysis indicates that higher levels of E2 priming promote a more elongated morphology in RAW 264.7 (male-derived) macrophages skewed toward an inflammatory phenotype, suggesting a shift toward a more proliferatively active and adherent state. Both cell lines demonstrated increased Ki67 expression, indicating enhanced proliferative capacity following inflammatory stimulation. Additionally, elevated E2 levels correlated with increased expression of the pro-inflammatory marker *iNOS* and decreased expression of the pro-regenerative marker *Arg-1* in both cell lines, supporting other observed phenotypic trends [36].

An imbalance between pro-inflammatory and pro-regenerative macrophage cell types is a common feature of many pathological conditions. For example, during wound healing, the initial acute inflammatory response is driven by the infiltration and differentiation of inflammatory macrophages. However, if this response fails to resolve, it can lead to chronic inflammation. Fluctuation in circulating E2 (such as across the menstrual cycle) has been attributed to exacerbated inflammatory symptoms in certain pathologies [37, 38]. Hormonal increases specifically have been implicated in the exacerbation and even the onset of certain chronic inflammatory states. For instance, in autoimmune rheumatic diseases (i.e. systemic lupus erythematosus) individuals are advised against hormone replacement therapies for E2 as this can exacerbate their autoimmune symptoms [39].

Additional observations from the phenotypic enzymatic analysis supports a broader mechanistic role for sex- and hormone-based factors in driving a greater pro-inflammatory phenotype and a dysregulated pro-regenerative macrophage response. Pro-inflammatory phenotypic capacity at the protein level was greater for male-derived murine macrophages relative to female-derived murine macrophages. iNOS production was at a detectable level for pro-inflammatory RAW 264.7 and increased with an increasing E2 presence, whereas iNOS production was undetectable in pro-inflammatory J774A.1 macrophages. This aligns with *iNOS* gene expression, which was downregulated relative to the calibrator in J774A.1 cells, but upregulated above the calibrator in RAW 264.7. These findings may help explain why, despite being less prevalent in males, certain autoimmune and cardiovascular diseases present with greater clinical severity in males compared to females [40, 41] Additionally, this may evidence that female cell types may exhibit some protective or resistant responses to inflammatory processes in the presence of changing E2 levels. In this study, + IL-4 J774A.1 macrophages demonstrated impaired *Arg-1* expression and MMP9 production at higher E2 dosing. This pattern may indicate a dysfunctional pro-regenerative phenotype. Impaired pro-regenerative macrophages, particularly those unable to effectively remodel the ECM, have been hypothesized to contribute to the initiation of autoimmune and chronic inflammatory conditions [42, 43]. This is likely due to their failure to clear cellular debris, which can promote autoantibody formation. These maladaptive immune responses appear to be influenced by both hormonal fluctuations and sex-based differences in estrogen signaling.

Our 3D culture studies revealed a significant reduction in proliferation when female-derived macrophages were exposed to a male-derived secretome, indicating that female-derived macrophage proliferation in 3D environments is critically dependent on sex-matched extracellular cues. One possible explanation of this observation is that the male-derived microenvironment contained elevated levels of pro-inflammatory factors—a common hallmark of male macrophages [44]. Male macrophages are generally more responsive to inflammatory stimuli and tend to produce higher levels of pro-inflammatory cytokines, such as TNFα [44]. This heightened inflammatory environment may have contributed to the observed suppression of female-derived macrophage proliferation in the sex-mismatched 3D condition. Interestingly, MMP9 analysis revealed a modest increase in MMP9 activity in RAW 264.7 macrophages when cultured in a sex-mismatched environment compared to sex-matched conditions. Although this change in MMP9 activity was not statistically significant, it remains notable, as MMP9 is an extracellular matrix (ECM)-remodeling enzyme typically associated with a pro-regenerative macrophage phenotype [45, 46]. Female macrophages have been known to secrete higher baseline levels of anti-inflammatory cytokines, such as interleukin-10 [44]. It is possible that the presence of these pro-regenerative soluble factors influenced the male-derived RAW 264.7 cells to modestly upregulate MMP9 activity.

These findings suggest that sex-mismatched conditions elicit divergent behavioral outcomes in male versus female-derived macrophages. Specifically, female-derived macrophages appear more sensitive to changes in proliferation, while male-derived macrophages demonstrate altered enzymatic activity (particularly through matrix remodeling enzymes like MMP9) when placed in a sex-mismatched context. These findings may also point towards differences amongst male and female macrophages' secretomic profiles that may be influenced by sex chromosomes, hormones, or other intrinsic differences [47, 48] Together, our data emphasize that both the sex of the macrophage and the sex of its surrounding environment play critical roles in shaping cellular behavior.

Several limitations of this study are acknowledged. Due to their consistency and availability, murine cell lines were utilized; however, future studies should incorporate primary and human-derived cells to enhance physiological relevance. Additionally, the macrophages used in this work originate from tumor-derived lines, which may not fully represent tissue-resident or peripheral blood mononuclear cell-derived macrophages. Future studies could include macrophage populations and subtypes from different tissue contexts which would offer deeper insight into how sex-based factors influence innate immune responses. While this study primarily focused on E2, it is also critical to examine the roles of other gonadal hormones, such as testosterone and progesterone, in modulating immune cell function. It is worth noting that E2 dosages utilized, although common throughout similar studies [2, 49] are supraphysiological. However, they are necessary to overcome experimental in vitro issues with estradiol cellular uptake [50], and overcome tight metabolic turnover/regulation [51, 52]. These concentrations also provide insight into non-homeostatic states, which are imperative when studying immunological dysregulations. This higher dosing approach also enables the observation of functional differences within an acute timeframe. Clinically, short-term hormone exposure has been shown to detectably skew long-term, hormone-based immunological programming in individuals undergoing gender-affirming hormone therapy [53]. It is important to acknowledge that the E2 concentrations used may not accurately reflect typical cell culture conditions, such as phenol additives or serum contributions. However, this approach allows for the examination and recognition of how these conditions may impact cell culture and the behavior of estrogen-responsive cells. Lastly, although hormone-stripping methods effectively isolate the effects of E2, they may also deplete other media components, which could influence experimental outcomes. Nonetheless, this approach strengthens the attribution of observed effects specifically to E2.

### Perspectives and significance

**Fig. 7:**
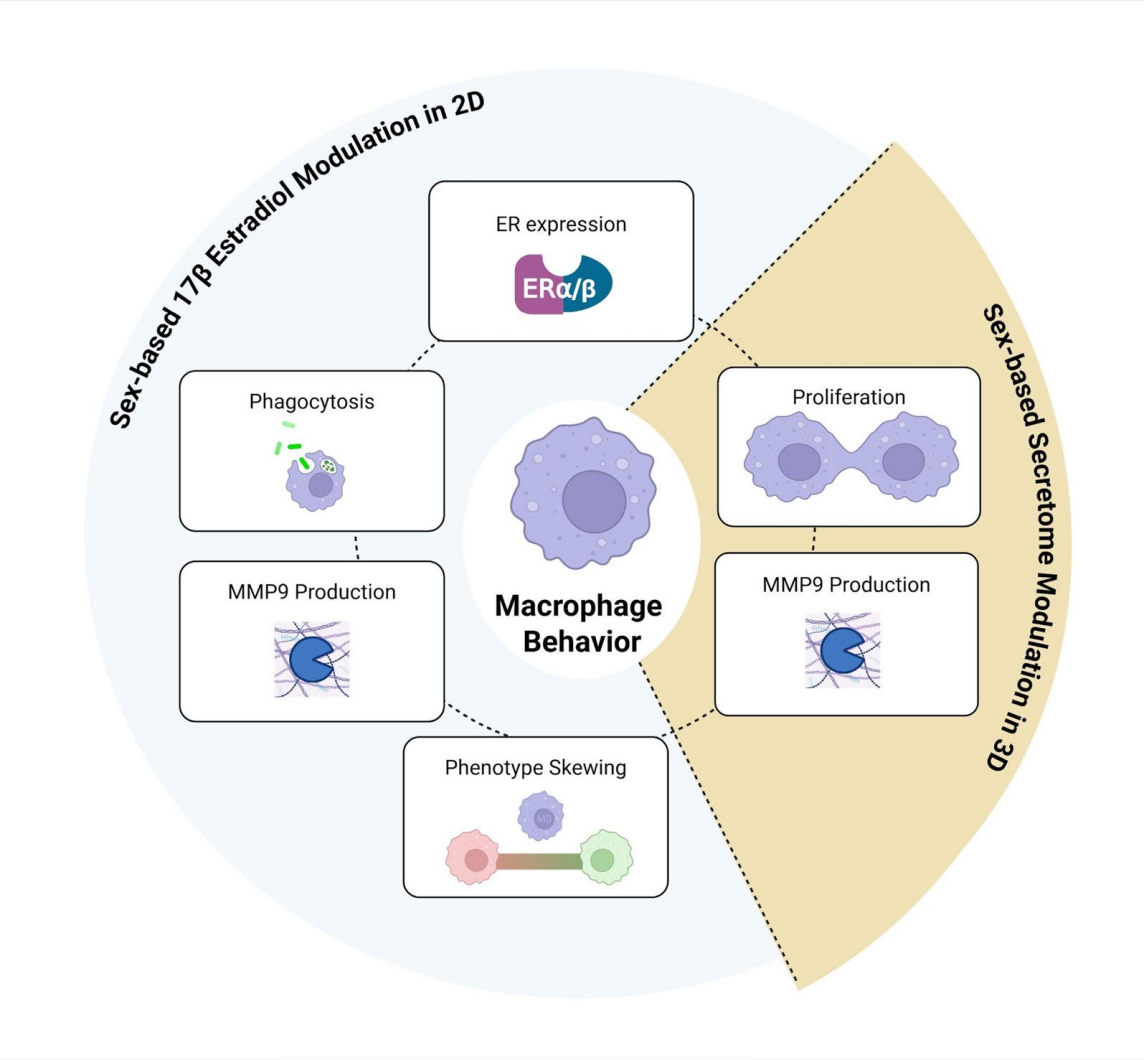
Summary of findings in investigation of sex-based factors influencing macrophage modulation

Our findings underscore the importance of considering sex when utilizing macrophages for in vitro studies. Although RAW 264.7 (male-derived) and J774A.1 (female-derived) cell lines are widely used in immunological research, their sex-specific responses remain poorly characterized. Here, we demonstrate that these two differently sexed cell lines present divergent proliferative behavior, phenotypic differentiation, and functional responses to E2 (Fig. 7). We also demonstrated that sex matching the culture environment is critically important in viability and ECM-related enzyme outcomes in our physiologically relevant 3D platform (Fig. 7). Overall, we implicated the influence of sex-based factors and hormonal environments in inflammatory biological biases. These differences highlight the necessity of creating sex-informed cell line selections and conditions to investigate immunological outcomes, particularly in hormone-responsive systems.

## Conclusions

This study provides evidence that sex-based differences and estrogen signaling critically shape macrophage behavior, from receptor expression to functional outcomes like phagocytosis, matrix remodeling, and inflammatory polarization. Sex is a complex variable that lacks universally defined metrics and is generally characterized by intrinsic factors, including XX or XY chromosomal karyotype and distinct hormonal profiles. Further research is needed to investigate how these defining characteristics influence sex-related immune responses. By characterizing the distinct responses of male- (RAW 264.7) and female-derived (J774A.1) macrophages to E2 and sex-matched or mismatched environments, we show that both the intrinsic sex of the cell and the surrounding extracellular context are fundamental drivers of hormone response and immune function. These findings reinforce the necessity of incorporating sex-accurate models into preclinical research workflows. Failure to do so may obscure key biological responses, contributing to translational inefficiencies and clinical blind spots—especially in inflammatory and hormone-responsive diseases. This work serves as a call to action for the integration of sex-informed design in in vitro systems, offering a foundational step toward more predictive, equitable, and effective diagnostic/modeling/therapeutic development.

## Supplementary Information

Additional file1

## Data Availability

No datasets were generated or analysed during the current study.
