# Supplementary material for: Cell line-specific estrogen responses uncover functional sex differences in murine macrophages
Source: Biol Sex Differ. 2025 Oct 14;16:75. doi: 10.1186/s13293-025-00760-1 (PMC12522439; doi:10.1186/s13293-025-00760-1)
Supplement: Supplementary file 1 — Additional file1 [file 13293_2025_760_MOESM1_ESM.docx]

Supplementary Materials for

**Cell Line-Specific Estrogen Responses Uncover Functional Sex Differences in Murine Macrophages**

Alison M Veintimilla, *et al.*

*Erika Moore corresponding author. Email: emt@umd.edu

| 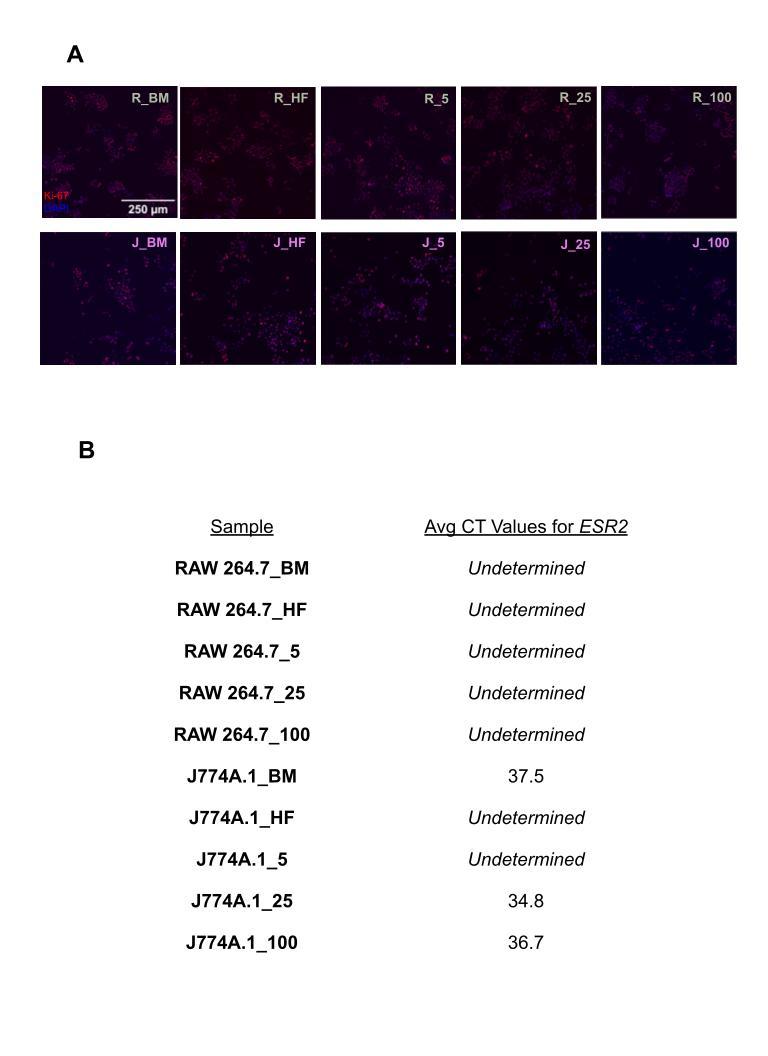 |
| --- |

**Fig. S1.**

E2 dosing does not have an effect on RAW 264.7 and J774A.1 proliferation. *ESR2* expression was not detected in either cell-line. (**A**) Representative images of immunocytochemistry staining for proliferation marker Ki67 (red), and cell-nuclear marker, DAPI (blue) on RAWs (top row - green) and J774s (bottom row - pink). **Scale bar showing 250 µm** (**B**) Threshold cycle (CT) values for *Esr2* expression - which encodes ERbeta - for RAW 264.7 and J774A.1. For figure and table, BM is referring to basal media conditions, HF is referring to hormone-free conditions and 5, 25,100, refers to E2 treatment in nM.


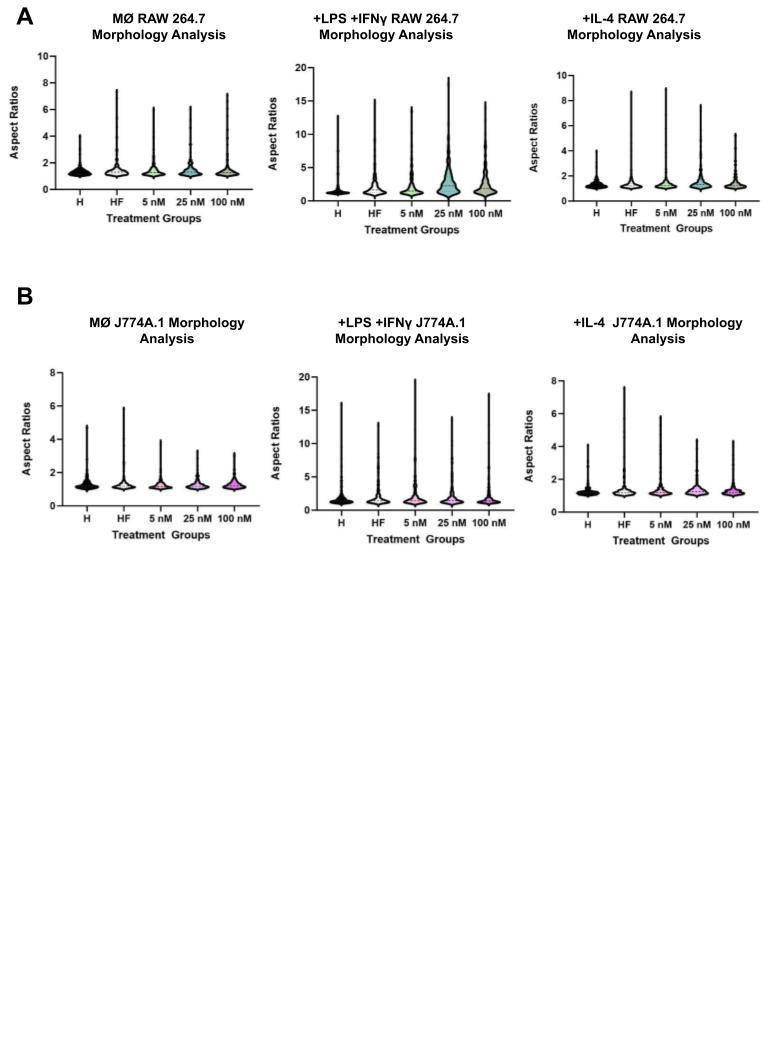


**Fig. S2.**

Aspect ratio distribution across +LPS+IFNγ and +IL-4 stimulated macrophages for RAW 264.7 (**A**) and J774A.1 (**B**).

| Sample | Avg CT Values for *iNOS* |
| --- | --- |
| **MØ RAW 264.7_BM** | *36.3* |
| **MØ RAW 264.7_HF** | *35.3* |
| **MØ RAW 264.7_5** | *36.3* |
| **MØ RAW 264.7_25** | *36.3* |
| **MØ RAW 264.7_100** | *36.0* |
| **MØ J774A.1_BM** | *32.4* |
| **MØ J774A.1_HF** | *34.8* |
| **MØ J774A.1_5** | *36.0* |
| **MØ J774A.1_25** | *34.3* |
| **MØ J774A.1_100** | *32.9* |
| **+IL-4 RAW 264.7_BM** | *38.2* |
| **+IL-4 RAW 264.7_HF** | *37.9* |
| **+IL-4 RAW 264.7_5** | *35.2* |
| **+IL-4 RAW 264.7_25** | *36.3* |
| **+IL-4 RAW 264.7_100** | *35.1* |
| **+IL-4 J774A.1_BM** | *34.6* |
| **+IL-4 J774A.1_HF** | *38.0* |
| **+IL-4 J774A.1_5** | *37.4* |
| **+IL-4 J774A.1_25** | *36.6* |
| **+IL-4 J774A.1_100** | *33.9* |

**Table S1.**

Table of cycle threshold (CT) values >/= 35 for *iNOS* gene expression for RAW 264.7 and J774A.1 across different macrophage phenotypes.

| Sample | Avg CT Values for *Arg-1* |
| --- | --- |
| **MØ RAW 264.7_BM** | *Undetermined* |
| **MØ RAW 264.7_HF** | *35.1* |
| **MØ RAW 264.7_5** | *Undetermined* |
| **MØ RAW 264.7_25** | *Undetermined* |
| **MØ RAW 264.7_100** | *Undetermined* |
| **MØ J774A.1_BM** | *Undetermined* |
| **MØ J774A.1_HF** | *35.3* |
| **MØ J774A.1_5** | *Undetermined* |
| **MØ J774A.1_25** | *37.4* |
| **MØ J774A.1_100** | *Undetermined* |

**Table S2.**

Table of CT values >/= 35 for *Arg-1* gene expression for RAW 264.7 and J774A.1 across different macrophage phenotypes.

**
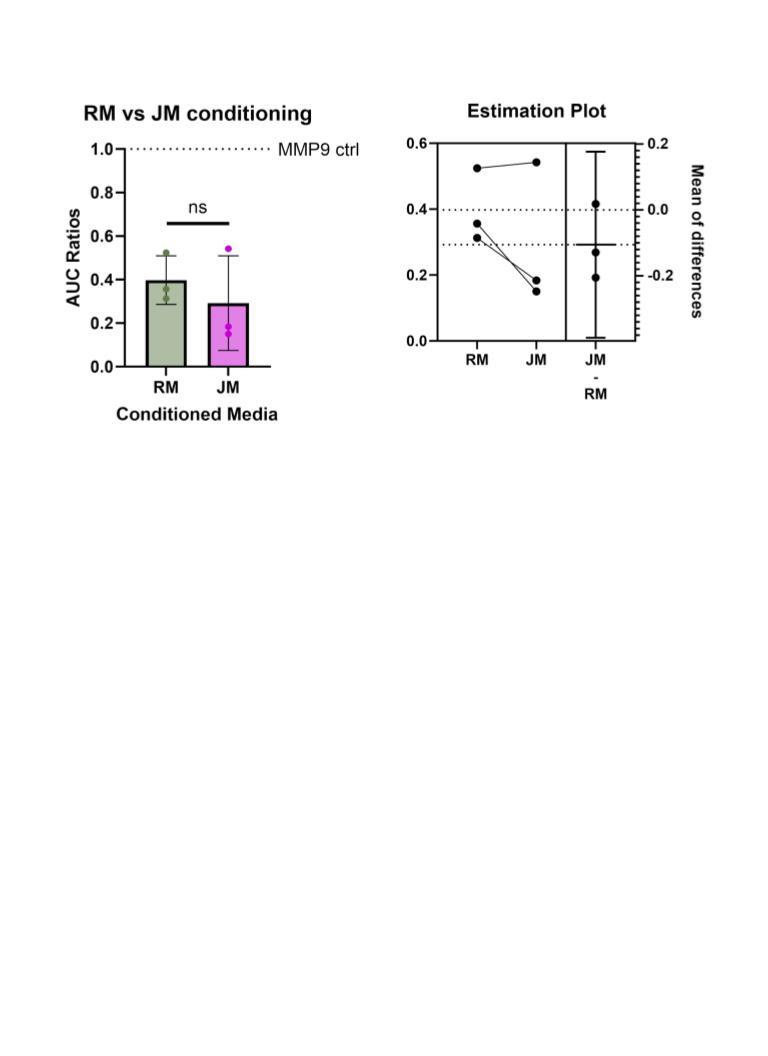
**

**Fig. S3.**

MMP9 quantification of RAW 264.7 and J774A.1 conditioned media with paired t-test statistical analysis estimation plot.
